# Effect of the Chinese traditional prescription Suo Quan Wan on TRPV1 expression in the bladder of rats with bladder outlet obstruction

**DOI:** 10.1186/s12906-015-0898-7

**Published:** 2015-12-01

**Authors:** Huanling Lai, Bo Tan, Zhijian Liang, Qitao Yan, Qingwang Lian, Qinghe Wu, Ping Huang, Hongying Cao

**Affiliations:** School of Chinese Materia Medica, Guangzhou University of Chinese Medicine, Guangzhou, 510006 China; School of Fundamental Medical Science, Guangzhou University of Chinese Medicine, Guangzhou, 510006 China; Research and Development department, The United Laboratories, Zhuhai, 528467 China

**Keywords:** TCM, Suo Quan Wan, TRPV1, OAB model rat, Overactive bladder, Urodynamic

## Abstract

**Background:**

Suo Quan Wan (SQW) is a Chinese traditional prescription that has been used in clinical treatment of lower urinary tract symptoms for centuries. However, scientific basis of SQW efficacy and mechanism is still needed. This study investigated the effect of SQW on bladder function and transient receptor potential vanilloid 1 (TRPV1) expression in the bladder of rats with bladder outlet obstruction (BOO). The induced changes in bladder function in overactive bladder (OAB) rat model were observed following different periods of outlet obstruction to obtain an appropriate rat model.

**Methods:**

This study was carried out in two parts. In the first part, female Sprague–Dawley rats received sham operations or partial BOO operations. Two, four, and six weeks later, the OAB model groups and control were subjected to urodynamic tests to measure differences in bladder functions. Once the appropriate rat model was obtained, the second part of the experiment was performed. The rat model was recreated and treated with SQW. Urodynamic assessment was conducted, and the bladders of the rats were then removed. Immunofluorescence staining, real-time PCR, and Western blot were performed to localize and quantify the expression of TRPV1 in the bladder.

**Results:**

Results of the first part indicated that at 2 and 4 weeks, the OAB model group exhibited significant differences in urodynamic parameters, including bladder pressure, maximum voiding pressure, and maximum bladder capacity, compared with the sham group. At 4 and 6 weeks, the OAB model group exhibited significant differences in residual volume (RV) and non-voiding contraction frequency. Six-week OAB model group showed much more RV but less voiding efficiency when compared with 6-week sham group or 2—and 4-week OAB model group. Rats that underwent BOO exhibited similarities with the compensated state before four weeks and may have entered decompensated state at six weeks. Studies conducted with 4-week OAB model were appropriate.

In part two of the experiment, unstable bladder in the OAB model group recovered bladder stability after SQW treatment, accompanied by improved bladder hypertrophy, as well as corrected urodynamic parameters. Expression of TRPV1 mRNA and proteins in the bladder was significantly greater in the OAB model group than that in the control group, which subsequently decreased significantly with SQW treatment in BOO-induced rats.

**Conclusions:**

SQW can modulate the expression of TRPV1 in accordance with the recovery of bladder function.

**Electronic supplementary material:**

The online version of this article (doi:10.1186/s12906-015-0898-7) contains supplementary material, which is available to authorized users.

## Background

The Standardization Sub-committee of the International Continence Society defines overactive bladder (OAB) as a syndrome characterized by urgency, with or without incontinence, usually with increased daytime frequency and nocturia in the absence of infection or other evident pathologies [1]. The symptoms of OAB include urge syndrome and urgency–frequency syndrome. Various factors and receptors in the bladder mediate these anatomic and functional changes. Stimulation of bladder afferent neurons by mechanical stretch of chemical irritation results in voiding responses that may be voluntary or involuntary in nature [2]. Of these, transient receptor potential vanilloid 1 (TRPV1) has been extensively studied.

TRPV1 is one of the most important and best understood representatives of the TRP family [3]. TRPV1 is a non-selective cation channel that may be activated by a wide variety of Internal and external physical and chemical stimuli, which has been cloned from rat dorsal root ganglia [4]. The urinary bladder is rich with vanilliod-sensitive afferent fibers that detect bladder distension or the presence of irritant chemicals, which in turn trigger reflex bladder activity. Clinically investigation has proved a novel approach in treating OAB-type symptoms by modulated of TRPV1 signaling [5]. TRPV1 is a pressure sensor in the bladder, mediating stretch detection [6]. Moreover, TRPV1 can also regulate urinary bladder contractions and Sensory function [7]. It plays a crucial role in maintaining bladder physiological function stability.

Herbals have been traditionally used for treatment of various diseases over centuries, including lower urinary tract symptoms (LUTS), such as OAB [8, 9]. The World Health Organization estimates that 80 % of the world’s population uses traditional medicine for primary health care. Women are more willingly to take complementary and alternative medicine [10]. In China, a wide range of Chinese medicinal herbs prescription have been used to improved or cured LUTS, such as nocturia, urgency, and child bedwetting. Suo Quan Wan (SQW) is one of the most commonly used traditional Chinese medicines (TCM) for treatment of various urinary system diseases in China, the first report of SQW was in the Southern Song Dynasty (between 1127 and 1279 CE) [11]. SQW is a mixture of *Alpinia oxyphylla* Miq., *Dioscorea opposita* Thunb., and *Radix Lindera* prepared in a ratio of 1:1:1, and is used to warm kidney yang and expel cold while relieving frequent urination by stopping leakage. SQW as a representative prescription of TCM therapeutic of nourishing the kidney and reducing urination by increasing the expression of AQP2 mRNA, AVPR-V2 mRNA [12] and CYP11B2 mRNA, increasing the level of Cort and ALD in blood accroding to the study of kidney deficiency polyuria rats [13]. SQW can also regulate the water metabolism and recovering the physiologic function of the detrusor in polyuria model animal [14]. Furthermore, SQW has significant effect on clinical treatment of OAB in China, but the mechanism remains unclear.

An appropriate animal model is essential to studies of pathology and mechanisms of OAB, along with drug effects. Various species have been used as animal models; the rat model induced via partial BOO is the most widely used. Experimental animal studies have demonstrated that the bladder progresses through three sequential stages (i.e., hypertrophy, compensation, and decompensation) in partial BOO [7]. The rat models are commonly assessed 2 [15] or 6 [16] weeks after BOO surgery. The best time to carry out the study is unclear.

We initially obtained an appropriate rat model by investigating the changes in bladder function in BOO rat models that are induced by outlet obstruction at different time points and examined using urodynamic tests. Appropriate rat model was used to further study the treatment mechanism of SQW in OAB. Considering that TRPV1 plays an important role in the bladder, including involvement in normal voiding function to pain sensation, this study conducted investigation about function and TRPV1 expression to clarify acting targets and pathway of SQW to OAB, as well as demonstrated the mechanism by which SQW interferes with TRPV1 expression of OAB.

## Methods

### Ethics statement

Protocols involved were in accordance to the rules and guidelines of the Experimental Animal Center of Guangzhou University of Chinese Medicine and were approved by the Guangzhou University of Chinese Medicine Animal Care and Use Ethics Committee (NO. 00066178, 2014/02/27-2014/04/03). And the performed of experiment was fit the international, national and institutional animal experiment rules. The rats were handled according to internationally accepted principles for the care and welfare of laboratory animals (E.E.C. Council Directive 86/609, O.J. no L358, 18/12/86). All the animal were sacrificed by anesthesia at the end of the experiment.

### Part 1 BOO rats model research

#### Animal grouping and surgical procedures

Fifty-four female Sprague–Dawley rats (200 ± 20 g) were obtained from Medical Experimental Animal Center of Guangzhou University of Chinese Medicine for the OAB rat model experiment. The rat license number was SCXK (YUE) 2013–0020. The rats were housed under a 12 h:12 h light/dark cycle at 20–24 °C, with free access to food and water for at least one week before the experiments were performed. Six groups were studied and consisted of sham-operated (2-, 4-, and 6-week groups; 8 animals per group) and BOO-operated (OAB model) (2-, 4-, and 6-week groups; 10 animals per group) animals.

To create the BOO model, we anesthetized female rats via intraperitoneal administration of 3 mL/kg pentobarbital. The bladder and proximal urethra were exposed via a lower abdominal midline incision. A 2–0 silk ligature was placed around the urethra and tied in the presence of an intra-luminally placed indwelling polyethylene cannula with an outer diameter of 1 mm. The abdominal wall was sutured after the polyethylene cannula was removed. Next, antibiotic medication (penicillin-G 400,000 I.U./kg) was administered. The obstructed rats underwent urodynamic tests at 2, 4, and 6 weeks after urethral ligature.

### Urodynamic test

Urodynamic evaluation was performed using a urodynamic measuring device (Laborite Delphis 94-R01-BT, Canada). Rats were anesthetized via administration of 10 % urethane (4.0 mg/kg) [17]. Polyethylene tubing with an outer diameter of 0.9 mm was inserted into the bladder through the urethra. The tubing was connected to a pressure transducer and a Harvard syringe pump using a three-way stopcock to record intra-vesical pressure and infuse saline into the bladder. After the bladder was emptied, cystometrography was performed via saline infusion at 0.2 mL/min. Maximum voiding pressure (MVP) and bladder pressure (BP) were then measured. Pumping was stopped as soon as urine was observed at the external orifice of the urethra. Bladder pressure obtained at this time was BLPP. Residual volume (RV) was measured by withdrawing intra-vesical fluid through the catheter. Maximum bladder capacity (MBC) was calculated as infusion speed multiplied by time. Voided efficiency (VE) was calculated as (MBC − RV)/MBC * 100 %. Bladder compliance (BC) was calculated as MBC/BLPP * 100. The number of non-voiding contractions (NVC) was measured during the filling period [18]. The values for the individual rats represent the means of two or three voiding cycles.

### Part 2 Effects of SQW on TRPV1 experession study

#### Drug preparation

In our study, the SQW was purchased from Hunan Hansen Pharmaceutical Co. Ltd. Briefly, the process and production are as follows, all these three components are weighed in the ratio of 1:1:1 and well-mixed after grinded into powder. Using appropriate distilled water to help these powder make into pills. According to the Chinese Pharmacopeia [19], assurance of quality control for SQW is validated and linderane is the recorded reference standard of SQW. HPLC and TLC were used to test these typical chemicals of SQW in our present experiment [20] (Data submitted as Additional file 1 and 2).

### Animal grouping and surgical procedures

After the appropriate rat model was selected, 84 were used to study the effects of SQW on TRPV1 expression under similar conditions. Five groups, namely, sham-operated group, OAB model group, SQW-treated low group (treated with SQW at 293 mg/kg/day), SQW-treated middle group (treated with SQW at 585 mg/kg/day), and SQW-treated high group (treated with SQW at 1170 mg/kg/day), were investigated. All animals were subjected to intra-gastric administration.

All animals received the same BOO-operated or sham-operated procedure. Drug administration was performed after 2 days and continued for 4 weeks.

### Urodynamic test

Urodynamic evaluation was performed following the former procedures. NVC was measured during the filling period and micturition times, and voided volume was recorded within 1 h after the last urodynamic test.

### Bladder harvesting and processing

All rats were sacrificed, and their bladders were excised at the bladder outlet and weighed [21]. The bladder was cut vertically and divided into three parts (one quarter for RT-PCR, two quarters for Western blot, and the rest for immunofluorescence staining). The portion used for RT-PCR and Western blot was stored in liquid nitrogen until needed. The portion used for immunofluorescence staining was embedded in optimum cutting temperature compound (Sakura, Japan) tissue freezing medium, quick-frozen in liquid nitrogen, and then stored at −80 °C until needed.

### Immunofluorescence staining

For immunofluorescence staining, the frozen tissue was sectioned at 12 μm. Bladder tissue slides were processed for routine immunohistochemistry. Frozen sections were fixed in cold acetone for 10 min. Slides were washed three times in PBS for 5 min and then blocked with 5 % BSA for 60 min. After incubation in blocking solution, the slides were incubated overnight at 4 °C with rabbit anti-TRPV1 and washed three times in PBS for 5 min. The tissues were then incubated with goat anti-rabbit IgG at 37 °C for 90 min and washed three times in PBS for 5 min. Tissues were mounted on slides and examined under laser scanning confocal fluorescence microscope (LSM 710; Carl Zeiss, Germany), at 488 nm.

### Real-time PCR

Total RNA from the bladder tissue was isolated by TRIZOL reagent and reverse transcribed into cDNA using RT-PCR kits (Thermo Fisher Scientific, USA) according to the manufacturer’s instructions [22]. The synthesized cDNA was amplified by quantitative RT-PCR on an ABI Prism 7500 system using SYBR Green RT-PCR master mix reagent (Thermo). Table 1 shows the expected RT-PCR product sizes and primers used in this study. The amplification cycle was 95 °C for 15 min, 40 cycles of 95 °C for 15 s, 60 °C for 30 s, and 72 °C for 30 s. Data were collected and analyzed by complementary computer software. Relative gene expression was calculated using the 2^−ΔΔCt^ method and normalized to GAPDH expression in each sample [23].

**Table 1:** Primers used for quantitative real-time polymerase chain reaction analysis of GAPDH and TRPV1

cDNA/product sizes	Sequence(5′-3′)
GAPDH	Forward primers: ggtgaaggtcggtgtgaacg
	Reverse primers: ctcgctcctggaagatggtg
TRPV1	Forward primers: gtttacctcgtccaccctga
	Reverse primers: agagagccatcaccatcctg

### Western blot

The tissue was homogenized, and total proteins were extracted using a total protein extraction reagent kit [24]. The protein concentration was measured by using a BCA protein assay kit (Pierce, USA). Protein samples were separated on SDS-PAGE gels at 70–90 V and transferred to PVDF membranes by using a transblotting apparatus (Bio-Rad Laboratories, USA) for 70 min at 90 V. The membranes were blocked with 5 % (w/v) non-fat milk at room temperature for 90 min and subsequently incubated overnight at 4 °C with rabbit anti-TRPV1 (1:1000; Abcam). The immune-labeled membranes were washed once with PBST for 15 min, followed by two separate washes (5 min/wash). The membranes were then probed with secondary antibody (1:2000; Millipore) at room temperature for 60 min in 5 % non-fat milk. The membrane was washed three times with PBST, and protein bands were visualized with ECL Western blotting detection reagents (Bio-Rad Laboratories, USA). The intensity of each target protein band was analyzed using an Image Station 4000R (KODAK, USA) and expressed relative to β-actin density.

### Data analyses

Data are expressed as means ± standard errors of mean. For multiple comparisons, repeated-measure ANOVA (Holm-Sidak) was used. Pairwise and non-pairwise comparisons were performed using Student’s *t*-test. Linear regression analyses were also utilized where appropriate, and ANCOVA was used to compare regression slopes and intercepts. The percentage of VE among groups was measured using Pearson chi-square test and presented as mean difference. These calculations were performed using SPSS 13.0 and were based on the number of individuals. *P <* 0.05 was considered statistically significant.

## Results

### Part 1 BOO rats model research

Pressures, volumes, and NVC frequencies are provided in Table 2. The 2- and 4-week OAB model groups exhibited significant increases in BP and MVP, as well as bladder capacity, compared with the corresponding sham groups. With regard to NVC, only 42 % (5 of 12 rats) of the 2-week OAB model group exhibited statistical differences compared with the sham group. However, in the 4-week OAB model group, 89 % (8 of 9 rats) exhibited significant increase in DO, as well as RV, compared with the sham group. After 6 weeks, the OAB model group exhibited an increase in RV and NVC, whereas voiding efficiency decreased significantly when compared with the 6-week sham group.

**Table 2:** Urodynamic variables at 2, 4, and 6 weeks after BOO

	N	Bladder pressure (p/mmH_2_O)	Max. voiding pressure (p/mmH_2_O)	Residual volume (V/ml)	Max. Bladder capacity (V/ml)	Voided efficiency %	Bladder compliance (ml/mmH_2_O)	Non-voiding contractions
2w sham	8	48.0 ± 2.2	49.7 ± 2.4	0.22 ± 0.02	0.90 ± 0.09	80	1.79 ± 0.22	0.08 ± 0.05
2WBOO	8	60.5 ± 2.0^##^	62.3 ± 2.2^##^	0.35 ± 0.09	1.68 ± 0.18^#^	76	2.27 ± 0.30	1.08 ± 0.49
4w sham	8	49.1 ± 1.5	50.4 ± 1.4	0.22 ± 0.03	1.04 ± 0.16	79	1.89 ± 0.33	1.06 ± 0.38
4WBOO	8	61.0 ± 3.0^##^	64.18 ± 3.0^##^	0.39 ± 0.06^#^	1.72 ± 0.22^#^	77	2.63 ± 0.44	6.60 ± 2.00^#^
6w sham	8	54.2 ± 0.6	55.3 ± 0.5	0.50 ± 0.09	1.78 ± 0.07	72	2.24 ± 0.12	1.62 ± 0.70
6WBOO	8	57.0 ± 2.1	58.2 ± 2.5	0.81 ± 0.17^#^	1.84 ± 0.26	55^#^	3.00 ± 0.47	3.85 ± 0.65^#^

Paired *t*-test: ^#^ *P <* 0.05; ^##^ *P <* 0.01. Student’s t-tests or Mann–Whitney U-tests when data were not normally distributed for comparisons between the BOO and sham groups at the same time points

**Fig. 1:**
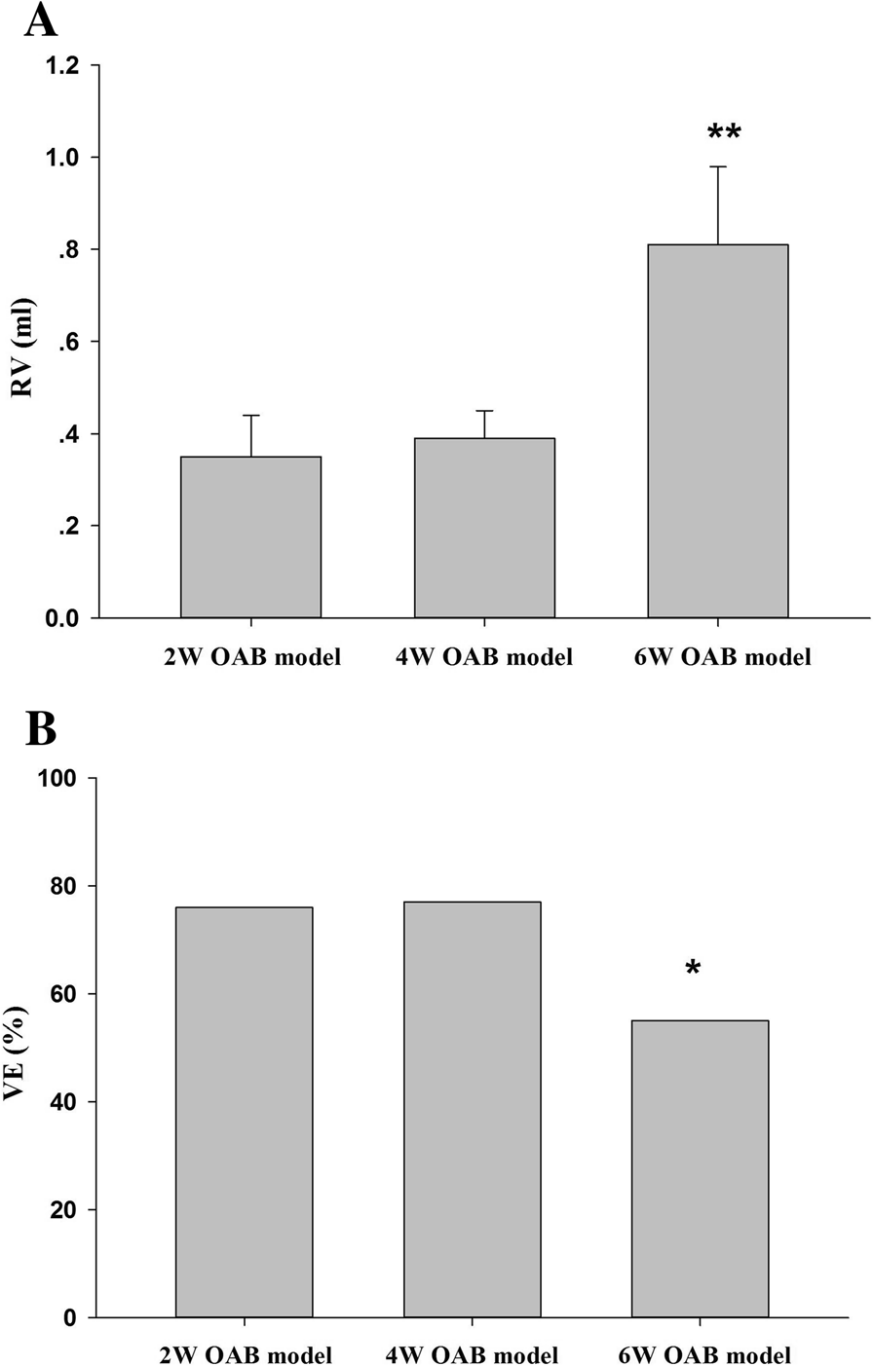
Comparisons of the residual volumes (RV) and vioding efficency (VE) comparisons of the OAB group at 2, 4 and 6 weeks. A: RV of 2, 4 and 6 weeks OAB group; B: VE of 2, 4 and weeks OAB group. Values are expressed as mean ± SEM. ★★ = *P <* 0.01, _★_ = *P <* 0.05 vs 2 and 4 weeks

Previous studies also compared urodynamic values among 2-, 4-, and 6-week OAB model groups. Pressures and volumes were similar between 2- and 4-week OAB model groups, but RV and NVC of the 4-week group were higher. Compared with 2- and 4-week OAB model group, RV of the 6-week OAB model group increased, whereas voiding efficiency decreased (Fig. 1a and b). Bladder pressures showed a slight decrease, but not statistically different. Moreover, the highest NVC frequency was observed in the 4-week group.

### Part 2 Effects of SQW on TRPV1 expression

**Fig. 2:**
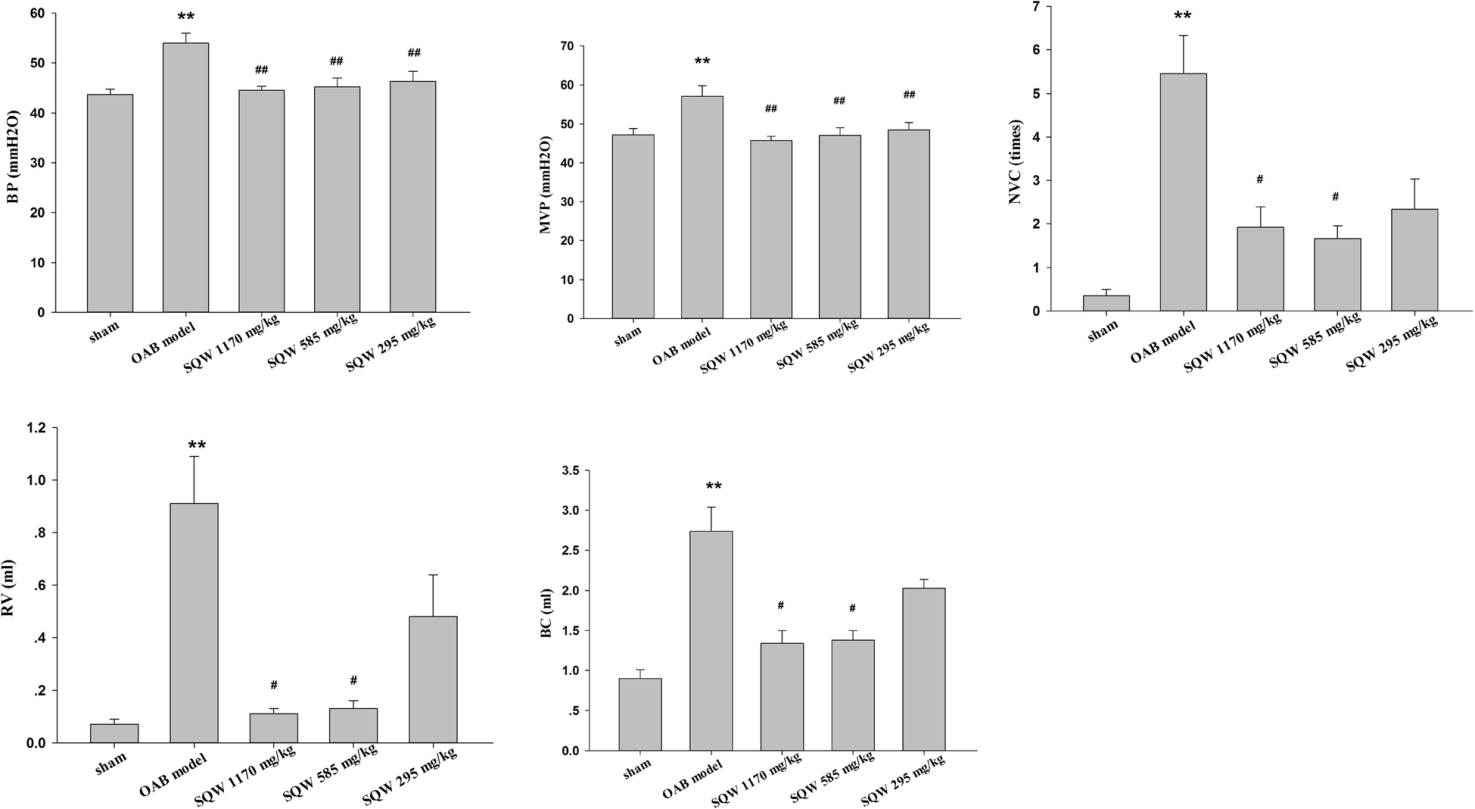
Urodynamic parameters of sham group, OAB model group, SQW treated groups (treated with SQW at 293, 585, 1170 mg/kg/day) are exhibited in the current figures, including bladder pressure (BP), maximum voiding pressure (MVP), maximum bladder capacity (MBC), residual volume (RV), non-voiding contraction (NVC). Values are expressed as mean ± SEM. ★ = *P <* 0.05, ★★ = *P <* 0.01 for comparisons between the OAB model group vs sham group. # = *P <* 0.05, ## = *P <* 0.01 for comparisons between the SQW treated group OAB model group

Based on the above mentioned studies, the 4-week OAB rat model was used to carry out the part 2 experiments. In accordance with previous experiment, the urodynamic results of OAB model groups showed significant increase in bladder pressures and capacity, NVC, and micturition frequency compared with sham group. SQW treatment with different dosages was beneficial to the OAB rat model, which exhibited significant decrease in bladder pressures and reduced MBC, RV, Vv, NVC, and micturition frequency dose dependently (Fig. 2).

**Fig. 3:**
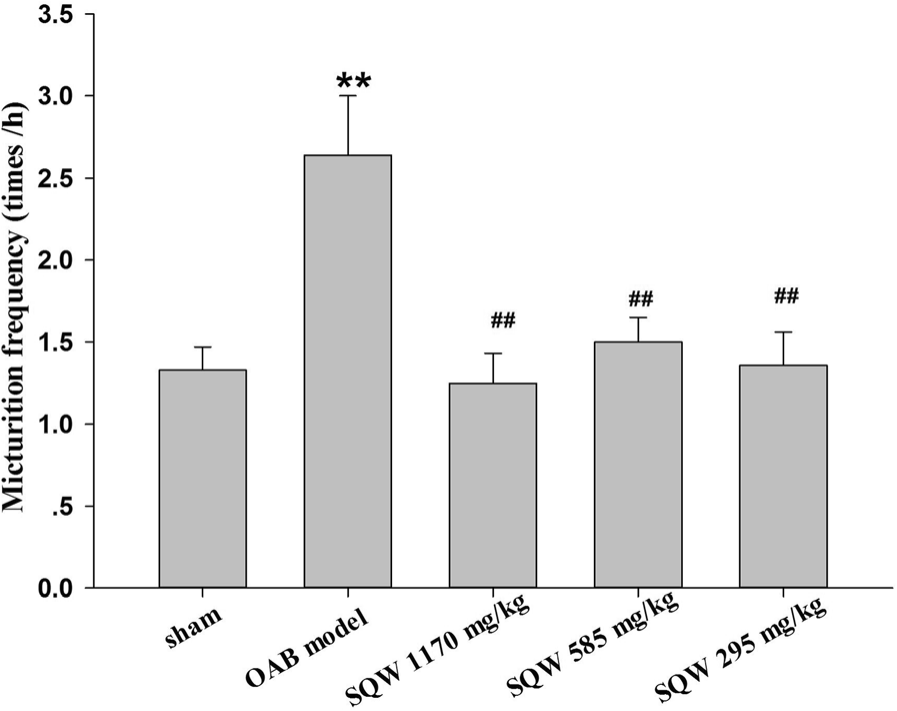
Micturition frequency of sham group, OAB model group, SQW treated groups (treated with SQW at 293, 585, 1170 mg/kg/day) were recorded within 1 h after the last urodynamic tests. Values are expressed as mean ± SEM. ★★ = *P <* 0.01 for comparisons between the OAB model group vs sham group. ## = *P <* 0.01 vs for comparisons between the SQW treated group OAB model group

**Fig. 4:**
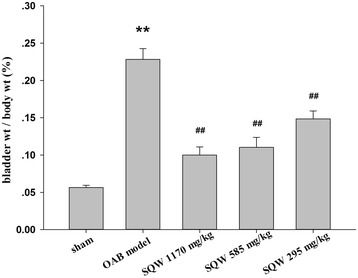
The ratio of bladder weight to body weight of sham group, OAB model group, tolterlodine treated group, capsazepine group (intravesical instillation at 10uM for 30 min before cystometrography), SQW treated group (treated with SQW at 293, 585, 1170 mg/kg/day). Values are expressed as mean ± SEM. ★★ = *P <* 0.01 for comparisons between the OAB model group vs sham group. ## = *P <* 0.01 for comparisons between the SQW treated group OAB model group

After urodynamic tests were conducted, bladder weight was measured, and the ratio of bladder weight to body weight was calculated. The ratio of bladder weight to body weight of OAB model rats was significantly increased (Fig. 3). Drug administration significantly reduced these indices and showed dose dependence in SQW treatment (Fig. 4).

**Fig. 5:**
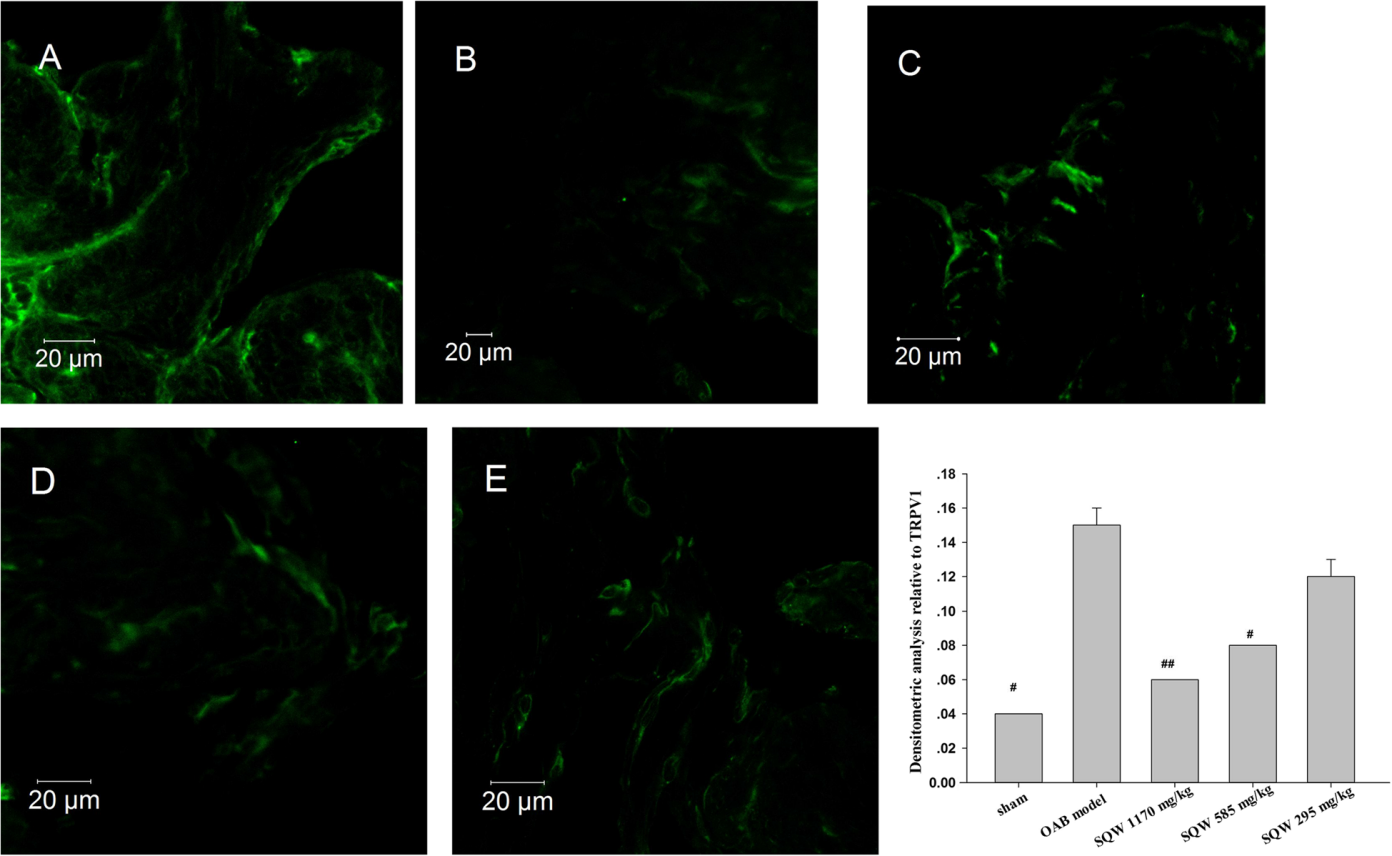
Inmunofluorescence staining used Laser Confocal Scan Analysis of TRPV1 in Bladder. **a**: OAB model group. **b**:sham group, **c**: SQW treated high group (treated with SQW at 1170 mg/kg/day), **d**: SQW treated middle group (treated with SQW at 585 mg/kg/day) and **e**: SQW treated low group (treated with SQW at 293 mg/kg/day). The last figure is densitomric analysis relative to TRPV1 of each group. Values are expressed as mean ± SEM. # = *P <* 0.05, ## = *P <* 0.01 vs OAB model group

**Fig. 6:**
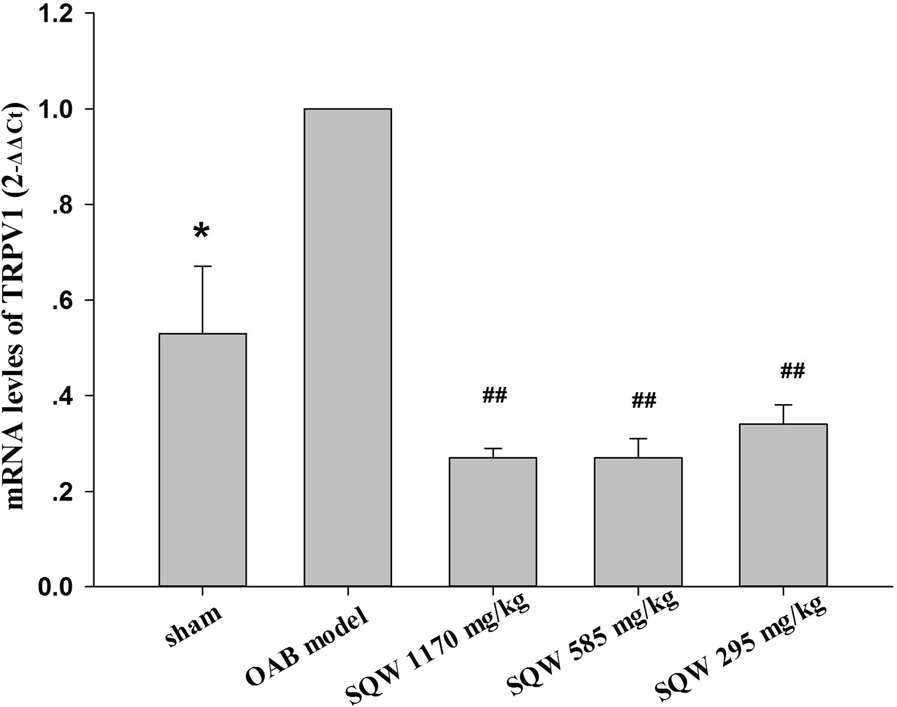
The effect of sham group, OAB model group, SQW treated group (treated with SQW at 293, 585, 1170 mg/kg/day) on TRPV1 mRNA expression in bladder. Values are expressed as mean ± SEM. ★ = *P <* 0.05, ## = *P <* 0.01 vs OAB model group

**Fig. 7:**
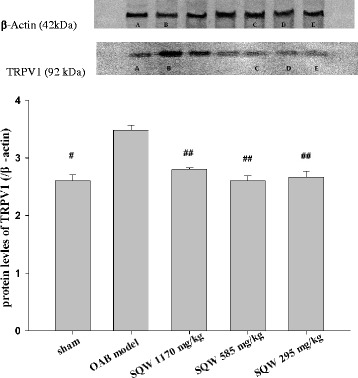
The effect of SQW treated on TRPV1 protein expression in bladder. A: sham group, B: OAB model group, C: SQW treated high group (treated with SQW at 1170 mg/kg/day), D: SQW treated middle group (treated with SQW at 585 mg/kg/day) and E: SQW treated low group (treated with SQW at 293 mg/kg/day). Values are expressed as mean ± SEM.# = *P <* 0.05, ## = *P <* 0.01 vs OAB model group

With regard to TRPV1 expression, immunofluorescence staining using scanning laser confocal analysis of bladder revealed that TRPV1 was localized in the bladder (Fig. 5). Densitometric analysis relative to TRPV1 showed that SQW could reduce TRPV1 expression in OAB rat bladder. RT-PCR and Western blot analysis demonstrated that OAB model increased the expression of TRPV1 mRNA and proteins in the bladder compared with the sham group (*P <* 0.01). Moreover, SQW treatment significantly reduced the expression of TRPV1 mRNA (Fig. 6) and proteins in the presence of OAB model (Fig. 7).

## Discussion

To the urinary bladder, the physiological function is store and periodic urination, and this function is regulated by the contraction and relaxation of the bladder wall which comprises by the detrusor smooth muscle [25]. As demonstrated in animal partial BOO experimental studies, the bladder undergoes three sequential stages, namely, hypertrophy, compensation, and decompensation [26].

Partial BOO induced rabbit OAB model results in increased bladder mass which induced by immediate increase in micturition pressure, smooth muscle hypertrophy, collagen synthesis and deposition, and urothelial and interstitial fibroblast proliferation [27, 28]. Previous studies showed that at 2 and 4 weeks after BOO, rats exhibit increased bladder pressure, and that during these periods, the animals require more pressure to empty the bladder, which leads to greater energy loss and results in increased RV. However, with regard to NVC frequency, the 2-week BOO group was not different from the sham group, indicating that during this period, the bladder enlarged to retain more urine and exhibited increased pressure to empty the bladder, but these processes did not lead to detrusor dysfunction [29]. This observation is similar to that of hypertrophy stage.

Right after that, the bladder progress in compensated stage; resistance to urine flow generation either remain stable or increases to greater than the control, and the empty ability of bladder decreases [30]. In the present study, most of the urodynamic parameters, including BP (which is related to the pressure at the beginning of micturition), MVP (which is related to the maximum bladder pressure during the micturition cycle), bladder capacity, and BC, of the 4-week BOO animals were similar to the 2-week animals. At 4 weeks, the OAB model significantly increased the RV and NVC compared with both the 4-week sham and 2-week OAB model groups. This finding indicates that the detrusor destabilized, which is similar to the shift from the compensated stage to the decompensated stage [31].

Without treatment, the compensated stage could be lasts for quite a long time then enters the final phase. During the decompensated stage, bladder function destabilize led to progressive decrease in both phasic contraction and tonic contraction response to stimulation, as well as progressive loss of the empty ability of bladder, an increase in RV as consequence [26]. Similar phenomena have been shown in previous studies. After 6 weeks of progression, the decrease in contraction exhibited by BOO animals may be due to energy deprivation [32]. RV significantly increased further, when compared to the 2- and 4-week OAB model animals, because of progressive energy loss. The above characteristics are similar to those of the decompensated stage. Detrusor overactivity subsequently occurs during intravesical instillation, and the transition from compensation to decompensation which is characterized by decreased detrusor contraction frequency in the non-voiding phase [26].

The BC values of the OAB model groups were not significantly different from those of the sham group during any of the three periods. However, lower values were observed in the sham groups [29]. Considering that the present study was designed with corresponding 2-, 4-, and 6-week sham groups, which were different from most previous studies, we made a comparison among the three sham groups. However, with the progression to six weeks, the sham-operated rats exhibited changes in pressure and volume but were not statistically different, suggesting that subsequent studies should include a corresponding sham group.

The present study revealed urodynamic changes that occur in rats in three different stages that follow partial BOO. Comparison of urodynamic results indicate that rats with BOO at four weeks were similar to the compensated stage and may enter the decompensated stage six weeks after BOO. The sham-surgery may negatively and progressively affect bladder function, which requires further research. Above all, rat model for OAB study was chosen before the decompensated stage, four weeks prior in our study.

Anticholinergics, specifically anti-muscarinic agents, are the most common medications prescribed for OAB. However, herbal treatments are an increasingly popular alternative for treating OAB [33]. The TRPV1 agonist capsaicin is also derived from herbal chili peppers; this agonist can bind with TRPV1 receptor, leading to reduction in neurotransmitter [34]. Results of previous studies exhibited that BOO rats have high expression of TRPV1 mRNA and proteins in the bladder. BOO rats exhibited increased bladder weight. Moreover, results showed increases in bladder pressures, capacity, non-voiding bladder contractions, and micturition frequency. These findings confirm the significant role of TRPV1 in bladder function, which is related to OAB.

The use of medicinal herbs has increased as part of complementary and alternative medicine [35]. Traditional prescription SQW has been used on clinical treatment of LUTS for decades and has shown to improve bladder function, as well as quality of life of OAB patients. Many potential targets exist in the treatment of LUTS because its pathophysiology is multifactorial [36]. SQW is composed of *A. oxyphylla* Miq, *Dioscorea rhizome* Thunb., and *Aconitii tuber* [the last two being ingredients in another Chinese herbal compound prescription Ba-Wei-Di-Huang-Wan (Hachi-mi-jio-gan)], which are speculated to possess a relaxant effect on the acetylcholine-induced contraction of smooth muscle and are used clinically for the treatment of LUTS [8]. In our study, OAB model rats treated with SQW exhibited decreased TRPV1 expression in the bladder, as well as reduced non-voiding bladder contractions and micturition frequency, indicating that SQW may have stabilizing effect on the excitability of bladder smooth muscle during filling related with neurogenic pathway via regulated TRPV1 expression. SQW also decreased bladder weight of OAB model rats; it also decreased bladder pressure, but improved micturition efficiency, which results in decreased RV. These results demonstrate that SQW can slow down the progress of OAB and improve overall bladder function, even the physical condition of animals.

Previous studies indicated that SQW treatment on OAB is related with TRPV1 regulation. Urothelium pathway may be involved in the mechanisms of SQW work through TRPV1 channel to adjust bladder function. Therefore, we aim to research on these mechanisms further.

## Conclusions

The main findings of the present study are as follows. First, the expression of TRPV1 increased in the bladder after induction of BOO, which decreased later in the SQW-treated group, compared with the OAB model group, and showed dose-dependent effects. Second, results of TRPV1 expression in the bladder are in agreement with urodynamic change, according to the induction of OAB model and SQW treatment. Furthermore, the outcome of the current study provides strong foundation to our hypothesis that treatment of SQW on OAB is related to TRPV1 and fulfills the scientific basis of the efficacy and mechanisms of SQW.

## Additional files

Additional file 1:
**The chemical characterization of SQW.** (PDF 295 kb) Additional file 2:
**A Chinese article about Suoquan Wan combined with Solifenacin Succinate on overactive.** (PDF 139 kb)
