# Supplementary material for: Effect of the Chinese traditional prescription Suo Quan Wan on TRPV1 expression in the bladder of rats with bladder outlet obstruction
Source: BMC Complement Altern Med. 2015 Dec 1;15:424. doi: 10.1186/s12906-015-0898-7 (PMC4666052; doi:10.1186/s12906-015-0898-7)
Supplement: Additional file 1: — The chemical characterization of SQW. (PDF 295 kb) [file 12906_2015_898_MOESM1_ESM.pdf]

·临床中药·

缩泉丸与琥珀酸索利那新联合治疗肾虚型膀胱过度活动症72例临床观察

蔡剑[1]，曾文彤[1]，张培海[1]，李广森[1]，夏雨果[1]，吴天浪[1]，彭成[2]

[摘要] **目的：** 观察中药缩泉丸汤剂联合琥珀酸索利那新治疗肾虚型膀胱过度活动症临床疗效。**方法：** 将72例肾虚型膀胱过度活动症患者随机分为2组，对照组36例予琥珀酸索利那新治疗，治疗组36例在对照组治疗基础上加用中药缩泉丸汤剂治疗。2组均服药6周。**结果：** 治疗组治疗后24 h平均排尿次数少于对照组(P＜0.05)，每次排尿量多于对照组(P＜0.05)。2组治疗后平均24 h尿失禁次数比较差异无统计学意义(P＞0.05)。治疗组不良反应率低于对照(P＜0.05)。**结论：** 缩泉丸联合琥珀酸索利那新治疗肾虚型膀胱过度活动症疗效确切。

[关键词] 膀胱过度活动症；肾虚；缩泉丸；琥珀酸索利那新

[中图分类号] R 285.6 **[文献标识码]** A **[文章编号]** 1674-926X(2013)05-013-03

Observation of the effect of Suoquan Wan combined with Solifenacin Succinate on overactive bladder caused by kidney deficiency/ *CAI Jian[1], ZENG Wen-tong[1], ZHANG Pei-hai[1], LI Guang-sen[1], XIA Yu-guo[1], WU Tian-lang[1], PENG Cheng[2] //(1.Department of Urology, Affiliated Hospital, Chengdu University of Traditional Chinese Medicine, Chengdu 610072, China;2.Pharmacy College,Chengdu University of Traditional Chinese Medicine;The Ministry of Education Key Laboratory of Standardization of Chinese Herbal Medicine;State Key Laboratory Breeding Base of Systematic Research, Development and Utilization of Chinese Medicine Resources, Chengdu 611137, China)*

[Abstract] **Objective:** To observe the therapeutic effect of Suoquan Wan combined with Solifenacin Succinate on overactive bladder. caused by kidney deficiency **Method:** 72 cases of overactive bladder were randomly divided into treatment group(n=36), which were treated by Suoquan Wan combined with Solifenacin Succinate and control group(n=36), which were given Solifenacin Succinate. The treatment continued for six weeks. **Result:** The average frequency of urination after treatment for 24 h was decreased with increased voided volume compared with control group (P＜0.05).There was no significant difference of the effect on the average 24 h urinary incontinence between two groups after treatment (P＞0.05).Adverse reaction cases in treatment group was less than that in control group (P＜0.05). **Conclusion:** Suoquan Wan combined with Solifenacin Succinate has positive effect on overactive bladder.

[Key words] Overactive bladder；kidney deficiency；Suoquan Wan；Solifenacin Succinate

膀胱过度活动症(overactive bladder，OAB)是泌尿外科常见病，表现为尿频、尿急和/或尿失禁，严重影响患者生活质量。目前中医药辅助治疗是常用手段，对改善OAB患者症状和体征有明显效果。2012年01月～2012年12月，我们运用中药缩泉丸联合琥珀酸索利那新治疗OAB肾虚型36例，琥珀酸索利那新治疗36例作为对照观察，结果如下。

1 资料与方法

1.1 病例选择

1.1.1 诊断标准 西医诊断标准参照“膀胱过度活动症临床指导原则”确诊[1]。中医诊断标准根据《实用中医内科学》“小便不禁”肾虚型确诊[2]。

1.1.2 纳入标准 ①符合以上诊断标准；②符合小便不禁肾虚型证型；③就诊前未进行过相关治疗，症状持续＞6月。签署知情同意书。

1.1.3 排除标准 24 h平均尿量＞200 mL或平均排尿次数＜8次者；怀孕、哺乳期妇女及拟怀孕的女性；膀胱出口梗阻者；明显压力性尿失禁者；明显肝肾功能异常者；泌尿系感染者；抗胆碱能药物禁忌者或过敏者；对中、西药治疗有选择和喜恶倾向者。

1.1.4 剔除标准 自动终止治疗或失去联系者；治疗

[作者单位] 1.成都中医药大学临床医学院，成都中医药大学附属医院泌尿外科，四川 成都 610072；2. 成都中医药大学药学院 中药材标准化教育部重点实验室 中药资源系统研究与开发利用省部共建国家重点实验室培育基地，四川 成都 611137

[作者简介] 蔡剑，博士在读，主要从事中药有效成分治疗泌尿外科疾病研究 Email:circleface1978@163.com

[通讯作者] 彭成，教授，博士生导师，主要从事疾病动物模型与中药复方药理毒理研究和中药新药开发

[收稿日期] 2013-03-18

万方数据

期间合并用任何其他抗胆碱能药物治疗者；未按治疗方案用药者；因药物不良反应而终止治疗者(不纳入疗效评价，但计入药物不良反应)。

1.2 一般资料

全部病例均为我院泌尿外科门诊患者，随机分为2组。治疗组36例，男16例，女20例；年龄19～68岁，平均(32.8±5.3)岁；病程6～24个月，平均(10 ± 0.5)个月。对照组36例，男17例，女19例；年龄19～72岁，平均(34.8±2.5)岁；病程6～22个月，平均(9 ± 1.5)个月。两组间一般资料比较差异无统计学意义(P＞0．05)，具有可比性。

1.3 治疗方法

2组均指导患者进行膀胱训练。

1.3.1 对照组　琥珀酸索利那新片(安斯泰来制药中国有限公司，国药准字J20090109)，5 mg/次，每日1次口服。

1.3.2 治疗组　在对照组治疗基础上加用中药缩泉丸汤剂(成都中医药大学附属医院中药房供药熬制)，100 mL/次，每日3次口服。

1.3.3 疗程　2组均6周为1个疗程，1个疗程后统计疗效。

1.4 观察项目

1.4.1 观察指标　①主观症状　包括患者尿急、尿频、尿失禁症状的改善情况及起效时间。症状改善程度分3 类：无受益、受益很少、受益很多。起效时间以天数记录。

②客观症状　24 h平均排尿次数及尿失禁次数的减少情况，平均每次尿量的增加情况。

病例记录由主管医师填写，记录患者的一般情况、病情变化及药物不良反应；患者填写治疗前后的排尿日记。

1.4.2 疗效评价　①主要疗效指标　平均每24 h排尿次数和平均每次排尿量。计算基线及治疗结束时、平均每24 h排尿次数和平均每次排尿量的均数；计算治疗结束时相对于基线的变化值。

②次要疗效指标　平均每24 h急迫性尿失禁次数,患者对治疗受益的主观感受和药物起效时间。

1.5 统计分析

应用SPSS 12.0软件进行统计学分析，均用双侧检验，P＜0. 05被认为差别有统计学意义。计数资料用χ2检验；计量资料用t检验(不符合参数检验条件则用秩和检验)。

2 结果

2.1 2组治疗前后平均24 h排尿次数、平均24 h尿失禁次数及平均每次排尿量比较，结果见表1。

表1　2组治疗前后平均24h排尿次数、平均24h尿失禁次数及平均每次排尿量比较（$\bar{x}\pm S$）

分 组	时间（周）	排尿次数 ($\bar{x}\pm S$)	尿失禁次数	排尿量 (mL)
治疗组（n=36）	0	13.2±3.5	2.2±1.3	132.8±22.5*
	6	6.8±2.7*△	0.8±0.5*	185.3±56.7*△
对照组（n=36）	0	10.4±4.2	2.0±1.5	128.4±17.5
	6	8.2±4.5*△	0.9±0.6*	165.2±39.3*△

与本组治疗前比较，*P＜0.05；与对照组治疗后比较，△P＜0.05。

由表1可见，2组治疗后平均24 h排尿次数、平均24 h尿失禁次数及平均每次排尿量与本组治疗前比较差异均有统计学意义(P＜0.05)；2组治疗后平均24 h排尿次数、平均每次排尿量比较差异均有统计学意义(P＜0.05)，治疗组疗效优于对照组。

2.2 患者主观疗效评价

结果显示，治疗6 周后，治疗组：19例(52.7%)受益很多, 11例(30.6%)受益很少，6例(16.7%)未受益；对照组：17例(47.2%)受益很多, 15例(41.7%)受益很少，4例(11.1%)未受益。2组50%的患者平均起效时间均为6天, 2组的主观受益人数和起效时间比较差异均无统计学意义(P＞0.05)。

2.3 不良反应情况比较

治疗组治疗过程中出现不良反应6例，不良反应率16.67% ；对照组出现不良反应10例，不良反应率27.78%。2组比较差异有统计学意义，治疗组低于对照组(P＜0.05)。

3 讨论

OAB是以尿频、尿急或急迫性失禁为特征的下尿路功能性疾病，目前估计全世界有5000万～1亿人口患有OAB[3]，其原因是非神经因素引起的膀胱逼尿肌不稳定。逼尿肌的收缩通过激动胆碱能M受体介导，故抗毒蕈碱制剂是治疗OAB的主要药物。琥珀酸索利那新是一种新型高选择毒蕈碱受体阻断剂，对毒蕈碱受体具有高亲和性和专一性。一项研究采用大鼠平滑肌和颌下腺模型检测本品对膀胱M型毒蕈碱受体的选择性，结果证实其对膀胱组织、膀胱内压的选择性比颌下组织和唾液腺更强，较其他毒蕈碱受体阻断剂更好[4]。但口干仍然是其主要不良反

万方数据

应。

祖国医学认为，小便不禁病症的发生与肾气的温煦和膀胱的固摄功能密切相关。《素问•宣明五气论》曰："膀胱不利为癃，不约为遗溺。"《诸病源候论•小便病诸候•小便不禁候》曰："小便不禁者，肾气虚，下焦受冷也。肾虚下焦冷，不能温制其水液，故小便不禁也。"现代医学认为，排尿功能主要涉及肾素-血管紧张素-醛固酮系统(RAAS)、下丘脑-垂体-肾上腺皮质(HPA)轴和醛固醇合成酶催化途径的调节功能。而RAAS系统、HPA轴与肾阳虚关系最为密切[5]。根据辨证病机为肾虚膀胱失约，故见尿频，温肾缩尿是治疗多尿行之有效的方法，主要通过影响RAAS系统和HPA轴发挥温肾、缩尿作用。缩泉丸由乌药、益智仁、山药组成，具有温肾祛寒、缩尿止遗、助膀胱气化的功效，主治肾阳虚所致的尿量增多、小便频数及遗尿。

本次临床观察发现，治疗组不良反应率低于对照组($P<0.05$)，可能与中药的综合调理作用有关。中医学认为，五液属津液范畴，皆由津液所化生，分布于五脏所属官窍之中，起着濡养、滋润以及调节津液代谢的作用。其中唾为肾之液，唾为肾精所化，肾精不能上承，口眼失于濡润则口干、眼干涩，不能上升清窍则头晕。缩泉丸可补肾生精摄津，从而减少口干的发生[6]。

在临床实践中，为提高疗效和减少琥珀酸索利那新不良反应，我们采用中药缩泉丸联合琥珀酸索利那新和膀胱训练的方法治疗OAB肾虚型病例，观察患者平均24 h排尿次数、平均每次尿量，治疗组疗效好于对照组($P<0.05$)，治疗后不良反应例数治疗组少于对照组，患者依从性较高，表明中药缩泉丸联合琥珀酸索利那新治疗OAB具有优势。

[参考文献]

[1] 金锡御,宋波,杨勇. 膀胱过度活动症指导原则[J]. 中华泌尿外科杂志,2002, 23(5):311.
[2] 王永炎,严世芸. 实用中医内科学[M].上海:上海科学技术出版社,1985; 307.
[3] Nowara A, Witek A, Wilk K. Diagnostic and treatment of overactive bladder[J]. Ginekol Pol, 2007, 78(7):549.
[4] J Ohtake A, Saitoh C, Yuyama H, et al. Pharmacological characterization of a new antimuscarinic agent, solifenacin succinate, in comparison with other antimuscarinic agents[J]. Z pharm Bu, 2007, 30(1): 54.
[5] 李淑雯,吴清和,黄萍. 缩泉丸对肾虚多尿大鼠肾素-血管紧张素-醛固酮系统的影响[J].中国实验方剂学杂志,2010,(11):108.
[6] 张雪松,成海生. 缩泉胶囊配合酒石酸托特罗定治疗膀胱过度活动症肾虚型36例临床疗效观察[J].河北中医, 2012,34(3):420.

(责任编辑：李芸霞)

（上接第31页）

证医学的思想和方法可在有毒中药控毒体系构建过程中得到充分应用并起到关键性作用，促进以循证为基础和特征的有毒中药控毒体系的建立与完善。

[参考文献]

[1] 彭成.中药药理学[M].北京:中国中医药出版社,2012.
[2] 孙守祥.木通药材基原考证[J].中药材,2007,30(7):875.
[3] 穆兰澄,高红.学习《伤寒论》中附子安全应用体会[J].辽宁中医药大学学报,2012,14(12),107.
[4] 高晓山,杨守业,何民,等.半夏妊娠禁忌的文献研究[J].中医杂志,1991, 12:44.
[5] 张文霞,钟希文.142例斑蝥中毒反应文献分析[J].时珍国医国药,2011,22(12):3042.
[6] 鹿树夏.误服天仙子中毒报告[J].吉林中医药,1988,1:21.
[7] 吴嘉瑞,张冰.鱼腥草注射液不良反应文献的数据挖掘研究[J].中国药物警戒,2007,4(6):360.
[8] 童元元,杨金生,李长虹,等.基于数据挖掘的中药安全性研究与思考[J].中国中药杂志,36(21):3048.

(责任编辑：蒋淼)

万方数据

万方数据 WANFANG DATA 文献链接

缩泉丸与琥珀酸索利那新联合治疗肾虚型膀胱过度活动症72例临床观察

作者：　蔡剑， 曾文彤， 张培海， 李广森， 夏雨果， 吴天浪， 彭成， CAI Jian， ZENG Wen-tong， ZHANG Pei-hai， LI Guang-sen， XIA Yu-guo， WU Tian-lang， PENG Cheng

作者单位：　蔡剑,曾文彤,张培海,李广森,夏雨果,吴天浪,CAI Jian,ZENG Wen-tong,ZHANG Pei-hai,LI Guang-sen,XIA Yu-guo,WU Tian-lang(成都中医药大学临床医学院,成都中医药大学附属医院泌尿外科,四川成都610072)， 彭成,PENG Cheng(成都中医药大学药学院 中药材标准化教育部重点实验室 中药资源系统研究与开发利用省部共建国家重点实验室培育基地,四川成都,611137)

刊名：　中药与临床

英文刊名：　Pharmacy and Clinics of Chinese Materia Medica

年，卷(期)：　2013,4(5)

参考文献(6条)

1. 金锡御;宋波;杨勇 膀胱过度活动症指导原则[期刊论文]-中华泌尿外科杂志 2002(05)

2. 王永炎;严世芸 实用中医内科学 1985

3. Nowara A;Witek A;Wilk K Diagnostic and treatment of overactive bladder 2007(07)

4. J Ohtake A;Saitoh C;Yuyama H Pharmacological characterization of a new antimuscarinic agent,solifenacin succinate,in comparison with other antimuscarinic agents 2007(01)

5. 李淑雯;吴清和;黄萍 缩泉丸对肾虚多尿大鼠肾素-血管紧张素-醛固酮系统的影响[期刊论文]-中国实验方剂学杂志 2010(11)

6. 张雪松;成海生 缩泉胶囊配合酒石酸托特罗定治疗膀胱过度活动症肾虚型36例临床疗效观察[期刊论文]-河北中医 2012(03)

引用本文格式：蔡剑.曾文彤.张培海.李广森.夏雨果.吴天浪.彭成.CAI Jian.ZENG Wen-tong.ZHANG Pei-hai.LI Guang-sen.XIA Yu-guo.WU Tian-lang.PENG Cheng 缩泉丸与琥珀酸索利那新联合治疗肾虚型膀胱过度活动症72例临床观察[期刊论文]-中药与临床 2013(5)
