# Supplementary material for: Effect of the Chinese traditional prescription Suo Quan Wan on TRPV1 expression in the bladder of rats with bladder outlet obstruction
Source: BMC Complement Altern Med. 2015 Dec 1;15:424. doi: 10.1186/s12906-015-0898-7 (PMC4666052; doi:10.1186/s12906-015-0898-7)
Supplement: Additional file 2: — A Chinese article about Suoquan Wan combined with Solifenacin Succinate on overactive. (PDF 139 kb) [file 12906_2015_898_MOESM2_ESM.pdf]

The quality control of Suoquanwan(SQW)

According to the Chinese Pharmacopeia, the content of linderane in SQW is required more than 0.09mg/g. And the our determination result show that the SQW we used is meet requirements.

Tab. The content of linderane in *Suoquan* pill

No.	Linderane(mg/g)
1	0.27
2	0.23
3	0.31
4	0.17
5	0.34

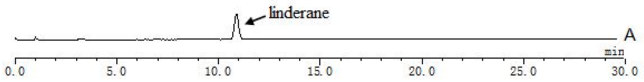

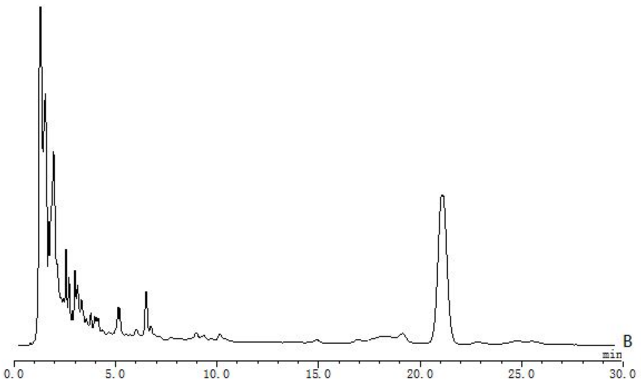

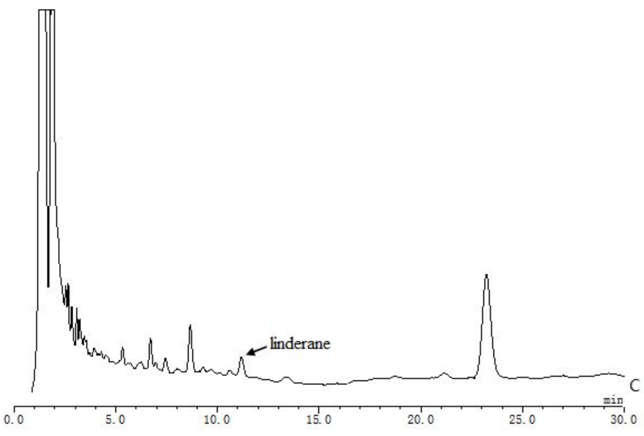

Fig. HPLC chromatography of SQW. Standard substance(A), Negative control(B), Chromatography of SQW(C)
